# High Blood Pressure and Depression Among the Working Population of Ghana: A Generalized Linear Model of the Risk Factors

**DOI:** 10.1155/2024/5261760

**Published:** 2024-09-30

**Authors:** Michael Arthur Ofori, David Kwamena Mensah, Ildephonse Nizeyimana, Niharika Jha, Zebunnesa Zeba, Shongkour Roy

**Affiliations:** ^1^School of Public Health, The University of Memphis, Memphis, Tennessee, USA; ^2^Department of Statistics, University of Cape Coast, Cape Coast, Ghana; ^3^University of Rwanda, Kigali, Rwanda

**Keywords:** depression, Ghana, high blood pressure, working population

## Abstract

**Background**. The global presence of high blood pressure and depression poses a significant public health threat, particularly in emerging nations. High blood pressure and depression are inevitable among the working population of Ghana, and it is crucial to recognize the potential influence of these conditions on the working-age population.

**Materials and Methods**. The study analyzed the risk factors associated with high blood pressure and depression among the working population of Ghana. The data in this study were drawn from Wave 1 data of the Study on Global Ageing and Adult Health (SAGE) survey, which was conducted by World Health Organization (WHO) in Ghana from January 2007 to December 2008. A longitudinal survey under the banner of SAGE was conducted. The study used 2681 participants aged 18–60 years. We modeled high blood pressure using logistic regression and depression with the proportional odds model of ordinal logistic regression.

**Results**. The study revealed that the prevalence of depression among the working-age population was 42.5%, whereas that of high blood pressure was 48.7%. The result also showed that males have a lower risk of developing high blood pressure and depression (OR = 0.851 and OR = 0.658, respectively) compared with females. Also, older adults (40–60 years) have a higher risk of developing high blood pressure and depression (OR = 1.992 and OR = 2.334, respectively) compared with younger adults. Other risk factors associated with high blood pressure include diabetes (2.107), depression, and weight. Last, alcohol intake (1.502), tobacco intake (1.279), and high blood pressure were found to be other risk factors associated with depression.

**Conclusion**. The prevalence of depression and high blood pressure is high among the working population of Ghana. There is therefore the need to incorporate health awareness programs on these topics.

## 1. Background

Globally, 1.3 billion people suffer from high blood pressure [1], and it is a significant health concern worldwide [2]. The prevalence of high blood pressure is predominantly observed in low- and middle-income countries [3, 4]. High blood pressure, also known as hypertension, is a significant health concern, among the working-age population of Ghana [5]. High blood pressure is a prevalent health issue in Ghana, affecting a significant portion of the adult population. While data may vary over time, it is a well-documented concern [6, 7].

High blood pressure is defined as a systolic blood pressure (SBP) of 130 or higher or a diastolic blood pressure (DBP) of 80 or higher [8]. Hypertension is a pathological state characterized by chronically elevated arterial blood pressure, which, if left unmanaged, can give rise to a range of adverse health outcomes. High blood pressure is not inevitable, and it is not a normal part of aging. Although high blood pressure is more common among older adults, it occurs in middle-aged and young adults too [9]. According to Lenon [9], high blood pressure affects around 7.5% of people aged 18–39 years, 33.2% of people aged 40–59 years, and 63.1% of individuals over the age of 60 years. Ghana has a youthful population. According to the 2021 population and housing census, ~60% of Ghana's population is of the working age (15–64 years) [10].

High blood pressure is a leading risk factor for cardiovascular diseases, stroke, and other related health problems like diabetes and depression [11, 12]. When high blood pressure is not effectively managed through lifestyle adjustments and medication, it can result in severe health complications, which may encompass cardiovascular conditions (such as heart disease and stroke), vascular dementia, vision issues, and kidney problems [8]. This will go a long way toward affecting productivity due to the nature of the population. If not managed properly, it may lead to absenteeism, reduced productivity, and an increased risk of disability and mortality.

Depression is also a risk factor for cardiovascular disease; it has some association with high blood pressure [13]. Depression is a common mental disorder. According to the World Health Organization (WHO), globally, an estimated 5% of adults suffer from depression [14]. In 2019, depression was the second-leading cause of disability globally [15]. It entails experiencing persistent feelings of sadness, reduced pleasure, or diminished interest in activities over extended periods. Depression stands apart from the typical mood fluctuations and common emotions associated with daily life. It can have an impact on various aspects of one's life, including their relationships with family, friends, and the community. Depression can affect individuals from all walks of life, and those who have endured experiences such as abuse, significant losses, or other sources of stress are more susceptible to developing depression. An estimated 3.8% of the world population is affected by depression, which includes 5% of adults, with 4% among men and 6% among women as well as 5.7% of adults over the age of 60. Approximately 280 million people in the world have depression [15] .

Depression is approximately 50% more prevalent in women compared to men [14]. Women exhibit a higher propensity to experience a greater quantity and intensity of stressful life events, including those influenced by genetic, biological, and environmental variables, in comparison to men [16, 17]. Another important issue, more than 700,000 individuals lose their lives to suicide annually, making it the fourth leading cause of death among individuals aged 15–29 years. The development of depression is the result of a complex interplay of social, psychological, and biological factors. People who have faced adverse life events such as unemployment, loss of loved ones, or traumatic experiences are at an increased risk of developing depression. Many of the factors influencing depression, such as a lack of physical activity or the harmful use of alcohol, are also recognized as risk factors for conditions like cardiovascular disease, cancer, diabetes, respiratory illnesses, and high blood pressure [14]. According to Schaare et al. [12], there is a stronger baseline association between SBP and better mental health among individuals who develop hypertension until follow-up.

Several factors contribute to high blood pressure and depression among Ghana's working population, including poor dietary habits, a lack of physical activity, obesity, excessive alcohol consumption, and stress. We also found that alcohol consumption and smoking were associated with higher SBP, DBP, depression, and hypertension among the middle-aged population. Although anyone can develop high blood pressure, men have a higher risk of doing so until the age of 45. From 45 to 64 years old, everyone has a similar risk of developing high blood pressure. On the other hand, older women have a higher risk of being depressed [18]. Men are more likely to develop high blood pressure before they reach the age of 55. Following the onset of menopause, women are more susceptible to experiencing elevated blood pressure. The chance of having high blood pressure increases as you get older, especially with isolated systolic hypertension [8]. Despite the increasing prevalence with age, certain lifestyle interventions can significantly reduce the risk of developing high blood pressure and depression. These include reducing salt intake, exercising regularly, stopping smoking, and eating a healthy diet [9].

Studies on high blood pressure in middle- and low-income countries concentrate on older adults leaving the working population, particularly in Ghana, which has a growing youthful population. This study aims to examine the prevalence of high blood pressure and depression and their associated risk factors among the working population (18–60 years) of Ghana.

## 2. Material and Methods

### 2.1. Data Source and Study Participants

Data for this study were drawn from Wave 1 data of the Study on Global Ageing and Adult Health (SAGE) survey collected in Ghana from January 2007 to December 2008 by the WHO. A longitudinal survey under the banner of SAGE was conducted in 2007 in six countries (China, Ghana, India, Mexico, the Romanian Federation, and South Africa) by the WHO regarding adults 18 years of age and older. The survey conducted on a sample of Ghanaian households employed a stratified multistage cluster sampling approach. First, a sampling method was used based on administrative division (using all 10 regions in Ghana) and nature of locale (urban or rural), generating 20 strata. Approximately 235 enumeration areas (EAs) were selected as primary sampling units from these localities, out of which 5259 households were surveyed. A total of 5573 (males = 2799 and females = 2764) respondents were then sampled from these households. Response rates at both household and individual levels were 86% and 80%, respectively. The data contain variables that enable the pursuance of this study, including variables on psychosocial factors, anthropometric factors, and sociodemographic characteristics. The study used 2681 participants aged 18–60 years.

### 2.2. Selected Variables

The study focused on high blood pressure and depression, and hence these two were considered the primary outcome variables of the developed models. Individuals who provided consent in the selected households participated in blood pressure measurements. The measurements were conducted three times using noninvasive techniques, employing a calibrated mercury sphygmomanometer. Each measurement was spaced 1–2 min apart, and the average of these three readings was recorded as the blood pressure of the respondent. WHO (2007–2010) measured the blood pressure of each respondent three times. It will be biased to use just any one of the three measurements and hence we took the average of the three measurements. For this study, we preferred to use the actual measurement and considered high blood pressure as having a mean SBP reading of at least 130/80 mmHg or higher. Also, respondents were asked if they had been diagnosed with depression, with the responses being yes (1) and no (0). Furthermore, participants were asked about the extent to which they experienced feelings of sadness, low spirits, or depression over the past 30 days as a single question. The responses were none (1), mild (2), moderate (3), severe (4), and extreme (5). For this study, depression was measured as a categorical variable using the second question, with responses of none (0), mild (1), and severe (2). Mild combines both mild (2) and moderate (3), while severe was made up of severe (4) and extreme (5). Other variables considered include age, sex, weight, marital status, pulse, alcohol intake, tobacco intake, rigorous exercise, and diabetes.

### 2.3. Logistic Regression Model

Assume *y* = [*y*_1_, *y*_2_,…, *y*_*t*_], *y*_*i*_ ∈ (0, 1), where *i* = 1,…, *t* with *y*_*i*_ = 1 representing the *i*th individual having a high blood pressure and *y*_*i*_ = 0 denoting *i*th individual without high blood pressure. Likewise, if we let *X*_*q*_ = [*X*_1_, *X*_2_,…, *X*_*q*_], *X*_*m*_ = [*x*_1*m*_, *x*_2*m*_,…, *x*_*qm*_], *m* = 1,…, *q* denote a *q*-dimensional predictor (sex, age, marital status, diabetes, weight, exercise, pulse, and depression status) vector predicting *y*. We also write *X* = [1, *X*_*q*_], where 1 denotes an *n* column vector of 1's for a *n* × (*q* + 1) design matrix, where we have written *a*′ as the transpose of the vector *a*. The success probability is then given by *p*(*y*_*i*_ = 1) = *τ* and that of failure by *p*(*y*_*i*_ = 0) = 1 − *τ*. Therefore, the model for generating the *y*_*i*_ is presumed to follow the Bernoulli probability model.

(1) $$\begin{array}{c} p\left(y_{i}\left|\tau \right.\right)=\tau^{y_{i}}{\left(1-\tau \right)}^{1-y_{i}},0<\tau <1. \end{array}$$

The set of predictors, *X*_*q*_ is then modeled using the logit link function:

(2) $$\begin{array}{c} \log\left(\frac{\tau_{i}}{1-\tau_{i}}\right)=X_{i}^{\prime }\beta , \end{array}$$

where $X_{i}=\left[1,x_{i1},x_{i2},\ldots ,x_{iq}\right],\tau =\frac{e^{X_{i}^{\prime }\beta }}{1+e^{X_{i}^{\prime }\beta }},1-\tau ={\left(1+e^{X_{i}^{\prime }\beta }\right)}^{-1},\text{ and }\beta =\left[\beta_{0},\beta_{1},\ldots ,\beta_{q}\right]$ represent the set a set of regression coefficients associated with the predictors.

### 2.4. Goodness of Fit Test

The likelihood ratio test of the hypothesis *H*_*o*_ : *c*(*β*) = 0, was used to assess the overall goodness of fit [19]. Here, we compared the likelihood that a respondent is depressed to the likelihood that he or she is not depressed. The deviance and Hosmer–Lemeshow (HL) tests were used to measure the fit of the model. The HL test tells whether the observed event rate matches that of the expected event rate in each subgroup [20]. It is given by:

(3) $$\begin{array}{c} G_{\text{HL}}^{2}=\sum_{i=1}^{10}\frac{{\left(o_{i}-e_{i}\right)}^{2}}{e_{i}\left(1-e_{i}/n_{i}\right)}∼\chi_{8}^{2}. \end{array}$$

### 2.5. Ordinal Logistic Regression Model

We considered the proportional odds model in this study. Generally, in the proportional odds model, if we assume that an ordinal outcome variable *W* (depression) has *Z* (none, mild, and severe) categories (*W* = 1, 2,…, *Z* − 1), then there exist *Z* − 1 distinct ways to dichotomize the outcome variable. That is, the odds of *W* ≥ *z* is given by:

(4) $$\begin{array}{c} \text{odds}\left(W\geq z\right)=\frac{p\left(W\geq z\right)}{p\left(W<z\right)}, \end{array}$$

where *z* = 1, 2,…, *Z* − 1. The assumption of proportional odds is very crucial as far as proportional odds model is concerned. The model assumes that the odds ratio assessing the effect of the response is the same regardless of where the cut-point is made. The dependence of the response on the predictor under the proportional odds model is given by:

(5) $$\begin{array}{c} \log\left[\frac{\eta }{1-\eta }\right]=\alpha_{z}+X_{i}^{\prime }\beta , \end{array}$$

where *η* = *p*(*y* ≥ *y*_*j*_ | *X*) is the cumulative probability of the event *y* ≥ *y*_*j*_, *X* = [1, *x*_1_, *x*_2_,…, *x*_*q*_], the regression coefficient of predictors *β* = [*β*_0_, *β*_1_,…, *β*_*q*_] and *z* = 1, 2,…, *Z* − 1.

### 2.6. Statistical Analyses

The study utilized both descriptive and inferential statistical analyses. The descriptive statistics were presented using tables with frequencies and percentages for categorical variables. A binary logistic regression was conducted to examine whether sociodemographic (gender, age, and marital status), self-rated health, comorbidities (depression and diabetes), and lifestyle (weight, regular exercise, smoking, and alcohol consumption) had a significant effect on the outcome variables (high blood pressure). Also, an ordinal logistic regression model was developed to examine the factors associated with depression among the working population. We used SAS 9.4 (SAS Institute, Inc, Cary, NC) to perform our statistical analyses, and we used a significant level of 0.05 for all the analyses.

## 3. Analysis

### 3.1. High Blood Pressure Model

High blood pressure is inevitable among the working population of Ghana.  Table 1 shows that 48.7% of the total sample of 2681 considered had high blood pressure. Among females, 603 (50.6%) out of 1191 have high blood pressure. The odds of a male having high blood pressure are 0.869 times the odds of a female having high blood pressure.

Additionally, 70.0% of respondents who have diabetes also have high blood pressure. High blood pressure and diabetes are not independent (*χ*^2^(1,  2681) = 13.083, *p* ≤ 0.001). The odds of a person with diabetes having high blood pressure were 2.517 times that of a person without diabetes having high blood pressure. Among the working population, age is still independent of high blood pressure, with those 40 years of age and older having a higher risk of developing high blood pressure (OR = 2.478).

Marital status and high blood pressure are also not independent (*χ*^2^(1,  2681) = 5.217, *p* = 0.022). Almost one-half (49.1%) of the respondents who are married, divorced, or separated have high blood pressure. There is a high risk of developing high blood pressure among non-singles (1.559) than among participants who were single.

The log odds of an individual within the working population of Ghana having a high blood pressure are dependent on several risk factors.  Table 2 reveals that among the risk factors that significantly contribute to high blood pressure among the working population are depression, gender, age, diabetes, pulse, and weight. The odds of a diabetic person having high blood pressure are 2.107 that of a nondiabetic person (OR = 2.107, *p* = 0.006). Likewise, the odds of an older person (40 years and older) having high blood pressure are 1.992 times that of a younger adult (18–39 years) having high blood pressure(OR = 1.992, *p* ≤ 0.001). In addition, men have a lower risk of developing a high blood pressure as compared to women. The odds of a man having high blood pressure are 0.851 times that of a woman.

There is a substantial improvement in fit in the final model as compared to the null model according to Table 3(*χ*^2^(8) = 122.436,  *p* ≤ 0.001). This is confirmed by the HL test (*χ*^2^(8) = 9.2177,  *p* = 0.3243).

### 3.2. Depression Model

Depression is also inevitable among the working population of Ghana. According to Table 4, 42.5% of the total sample of 2681 are depressed to some extent (either mild or severe). Among females, 539 (45.3%) out of 1191 are depressed. It is a little different among the males; 599 out of 1490 (40.1%) are depressed. There is a statistical association between sex and depression (*χ*^2^(2,  2681) = 14.796,  *p* ≤ 0.001). Again, age and depression are not independent. Among those aged 40 years and older, 1012 out of 2207 (45.9%) have some form of depression. This is very different among those who are less than 40 years old (26.6%; 126 out of 474). There is an association between age and depression (*χ*^2^(2,  2681) = 59.322,  *p* ≤ 0.001).

Also, Table 4 reveals that alcohol and tobacco intake have significant association with depression. Among those who take alcohol, 745 out of 1614 (46.2%) are depressed as compared to those who do not take alcohol (36.8%; 393 out of 1067). Alcohol intake and depression are not independent (*χ*^2^(2,  2681) = 23.475,  *p* ≤ 0.001). Likewise, among those who take tobacco, 270 out of 285 (47.8%) are depressed compared to those who do not take tobacco (40.9%; 853 out of 2085). Tobacco intake and depression are not independent(*χ*^2^(2,  2681) = 11.383,  *p* = 0.003).

Marital status and depression may have some association, but their association is not significant at the 5% significant level (*χ*^2^(2,  2681) = 5.333,  *p* = 0.0695). In addition, diabetes status has no association with depression (*χ*^2^(2,  2681) = 2.346,  *p* = 0.310).

We modeled depression status (none, mild, and severe) using the ordinal logistic regression. The proportional odds assumption was satisfied (*χ*^2^(6,  2681) = 7.8694,  *p* = 0.2478). This allowed us to use the proportional odds model of the ordinal logistic regression.  Table 5 presents the parameter estimates and the associated odds ratios.

The risk factors associated with depression among individuals within the working population of Ghana includes, but not limited to, sex, age, high blood pressure, tobacco, and alcohol intake. Males compared with females decrease the log odds of being at a higher level of depression by 0.4180. The odds of a male being severely depressed are 0.658 times that of a female (OR = 0.658,  *p* ≤ 0.001). Individuals who are 40 years and older are expected to increase the log odds of being at a higher level of depression by 0.8477. Age increases the risk of depression, and it is higher among older adults than young adults (OR = 2.234,  *p* ≤ 0.001). Tobacco intake and alcohol also increase the log odds of being at a higher level of depression by 0.2458 and 0.4068, respectively. Alcohol (OR = 1.505,  *p* ≤ 0.001) and tobacco (OR = 1.279,  *p* = 0.016) were found to increase the risk of depression among the working population. Marital status was not found to be a statistically significant risk factor for depression (OR = 1.030,  *p* = 0.8910).

## 4. Discussion

The findings of the study, which used Wave 1 data from the SAGE, suggest that high blood pressure and depression are associated risk factors among the working-age population (18–60 years) of Ghana. In developing nations, high blood pressure and depression represent a serious hazard to public health. Ghana has a youthful and most of them fall within the working age group (18–60 years). Even though high blood pressure and depression have historically been linked to aging, their effect on the working population can not be ignored. We found the prevalence of depression among the working population was 42.5%, whereas that of high blood pressure was 48.7%. These figures are higher as compared to the global average and other results reported in some studies. Mills et al. [4] in their systematic meta-analysis on global disparities of hypertension prevention and control from 90 countries, found that 31.1% of adults in the world suffer from hypertension. Among these, 31.5% are from low- and middle-income countries, while 28.5% are from high-income countries. Albasara et al. [21] found the prevalence of depression to be 19.6% among hypertensive patients in the Kingdom of Saudi Arabia. Li et al. [22] found the prevalence of depression among hypertensive patients to be 26.8%.

Age is one risk factor associated with both high blood pressure and hypertension. Most of these results come from studies that used the elderly population [23]. The result of the current study, which included the working-age population, was likewise consistent with earlier findings. Cheung et al. [24] found that the “score in the depression subscale (HADS-D) correlated with age and hypertension”. The study also found sex, weight, and diabetes to be risk factors for high blood pressure. Diabetes results in kidney scarring, leading to the retention of salt and water, subsequently elevating blood pressure. Over a period, diabetes harms the tiny blood vessels, causing them to stiffen and malfunction, contributing to increased blood pressure [25]. Dai et al. [6] concluded that the prevalence of hypertension increased with overweight, and this support our current result. Mozaffarian et al. [18] asserted that anyone can develop high blood pressure. They specified that men have a higher risk of doing so until the age of 45 years, yet from 45 to 64 years, everyone has a similar risk of developing high blood pressure. We found that men who are less than 40 years old have a lower risk of having high blood pressure compared to women, which is inconsistent with the assertion by Mozaffarian et al. [18]. The current study revealed that depressed men who are 40 years and above have higher risk of developing high blood pressure. The result is associated with other factors such as diabetes status, weight, and pulse (Table 2). The impact of gender on depression was not different from that of high blood pressure. We found that women have a higher risk of being depressed compared to men. This confirms the WHO's assertion that women are more likely to have depression than men [14]. The result was also not different from the conclusion by Mozaffarian et al. [18] that women have a higher risk of being depressed.

There is an established base-line association between depression and high blood pressure. In our logistic regression model, depression was found to be a risk factor for high blood pressure. Again, we found high blood pressure to be a risk factor for depression in the ordinal logistic regression model. These findings confirm the reports from some studies. Rubio-Guerra et al. [26] found that depression was common among uncontrolled hypertensive patients, which was approximately nine times that of the general population. They found a high prevalence of depression in hypertensive patients; this prevalence was approximately nine times greater than what is observed in the general population. In 1983, Rabkin, Charles, and Kass [27] found the interrelationship that exists between hypertension and depression. In their study, which involved 452 psychiatric outpatients with depression, they concluded that the prevalence of hypertension among those depressed was three times higher than that of nondepressed group [27]. Recent studies continue to show this association between depression and high blood pressure. High blood pressure can affect mental health and ultimately contribute to the development of hypertension and depression [12].

The study also found that alcohol and tobacco intake are risk factors for depression. Vallée [28] found a similar result and asserted that smoking and drinking are associated with higher SBP, DBP, depression, and hypertension among middle-age population. Although depression and high blood pressure have been linked to old age, certain lifestyles, like alcohol drinking, use of tobacco products, as well as lack of exercise, have been linked to depression. As a result, Lennon [9] instigate that certain lifestyle interventions can significantly reduce the risk of developing high blood pressure and depression, including reducing salt intake, exercising regularly, stopping smoking, and eating a healthy diet. According to the National Institute on Aging [8], high blood pressure needs to be managed effectively through lifestyle adjustments and medication in order to hinder its health complications, which may encompass cardiovascular conditions, dementia, vision issues, and depression.

## 5. Conclusions

The study reveals that high blood pressure and depression significantly affect the working population in Ghana, with age, sex, depression status, weight, diabetes, and alcohol intake as risk factors. Employers should incorporate health awareness talks and screenings for high blood pressure and depression into their yearly on-the-job training sessions.

## Figures and Tables

**Table 1:** A cross tabulation showing the association between high blood pressure and some selected variables.

Variables		High blood pressure (*N*, %)		Total	*χ* ^2^	*p* value	Odds ratio
		No (0)	Yes (1)				
Total	—	1376 (51.3)	1305 (48.7)	2681	—	—	—
Sex	Female (R)	588 (49.4)	603 (50.6)	1191	3.275	0.070	0.869
	Male	788 (52.9)	702 (47.1)	1490			
Diabetes	No (R)	1355 (51.9)	1256 (48.1)	2611	13.083	≤0.001	2.517
	Yes	21 (30.0)	49 (70.0)	70			
Age	<40 (R)	321 (67.7)	153 (32.3)	474	13.083	≤0.001	2.291
	≥40	1055 (47.8)	1152 (52.2)	2207	61.971	≤0.001	
Marital status	Single (R)	71 (61.7)	44 (38.3)	115	5.217	0.022	1.559
	Not single	1305 (50.9)	1261 (49.1)	2566			
Exercise	No (R)	1328 (51.5)	1253 (48.5)	2681	0.459	0.4979	1.148
	Yes	48 (48.0)	42 (52.0)	100			
Depression	No (R)	776 (50.3)	767 (49.7)	1543	1.551	0.213	0.907
	Yes	600 (52.7)	538 (47.3)	1138			

**Table 2:** Summary of maximum likelihood estimates of logistic regression model (2).

Parameter	Estimate	Error	Wald *χ*^2^	Pr > *χ*^2^	OR	95% CI of OR	
Intercept	−3.2613	0.3861	71.3306	≤0.001	—	—	—
Depressed (yes)	−0.2356	0.0814	8.3769	0.0038	0.790	0.674	0.927
Sex (male)	−0.1608	0.0803	4.0136	0.0451	0.851	0.727	0.997
Age (≥40)	0.9074	0.1115	66.2696	≤0.001	1.992	1.083	2.478
Marital status (not single)	−0.0836	0.2191	0.1455	0.7028	0.920	0.599	1.413
Pulse	0.0149	0.0030	25.4472	≤0.001	1.015	1.009	1.021
Weight	0.0043	0.0021	4.2349	0.0396	1.004	1.000	1.008
Exercise (yes)	0.4310	0.2173	3.9345	0.0473	1.539	1.005	2.356
Diabetes (yes)	0.7451	0.2699	7.6203	0.0058	2.107	1.241	3.576

**Table 3:** Model fitting information.

Test	*χ* ^2^	DF	Pr > *χ*^2^
Likelihood ratio	122.4363	8	≤0.001
Score	117.3399	8	≤0.001
Wald	108.9222	8	≤0.001
Hosmer–Lemeshow	9.2177	8	0.3243

**Table 4:** A cross tabulation showing the association between depression and some selected variables.

Variables		Depression			Total	*χ* ^2^	*p* value
		None (0)	Mild (1)	Severe (2)			
Total	—	1543 (57.5)	1056 (39.4)	82 (3.1)	2681	—	—
Sex	Female (R)	652 (54.7)	488 (41.0)	51 (4.3)	1191	14.796	≤0.001
	Male	891 (59.8)	568 (38.1)	31 (2.1)	1490		
Diabetes	No (R)	1507 (57.7)	1026 (39.3)	78 (3.0)	2611	2.346	0.310
	Yes	36 (51.4)	30 (42.9)	4 (5.7)	70		
Marital status	Single (R)	78 (67.8)	35 (30.4)	2 (1.7)	115	5.333	0.0695
	Not single	1465 (57.1)	1021 (39.8)	80 (3.1)	2566		
Exercise	No (R)	1476 (57.2)	1026 (39.8)	79 (3.1)	2580	3.936	0.140
	Yes	67 (67.0)	30 (30.0)	3 (3.0)	100		
Alcohol intake	No (R)	674 (63.2)	368 (34.5)	25 (2.3)	1067	23.475	≤0.001
	Yes	869 (53.8)	688 (42.6)	57 (3.5)	1614		
Age	<40 years	348 (73.4)	117 (24.7)	9 (1.9)	474	59.322	≤0.001
	≥40	1195 (54.1)	939 (42.6)	73 (3.3)	2207		
Tobacco intake	No (R)	1232 (59.1)	786 (37.7)	67 (3.2)	2085	11.383	0.003
	Yes	311 (52.2)	270 (45.3)	15 (2.5)	596		

**Table 5:** Summary of maximum likelihood estimates of the ordinal logistic regression model (5).

Parameter	Estimate	Error	Wald *χ*^2^	Pr > *χ*^2^	OR	95% CI of OR	
Intercept (3)	−4.2308	0.2433	302.2919	≤0.001	—	—	—
Intercept (2)	−1.0189	0.2174	21.9687	≤0.001	—	—	—
HBP (yes)	−0.1998	0.0798	6.2715	0.0123	0.819	0.700	0.958
Sex (male)	−0.4180	0.0864	23.4066	≤0.001	0.658	0.556	0.780
Age (≥40)	0.8477	0.1197	50.1799	≤0.001	2.334	1.846	2.951
Marital status (not single)	0.0298	0.2177	0.0188	0.8910	1.030	0.672	1.578
Tobacco intake (yes)	0.2458	0.1023	5.7745	0.0163	1.279	1.046	1.563
Alcohol intake (yes)	0.4068	0.0839	23.5366	≤0.001	1.502	1.274	1.770

## Data Availability

The data are available on request.
